# Supplementary material for: Targeted Disruption of the MORG1 Gene in Mice Causes Embryonic Resorption in Early Phase of Development
Source: Biomolecules. 2023 Jun 24;13(7):1037. doi: 10.3390/biom13071037 (PMC10377003; doi:10.3390/biom13071037)
Supplement: Supplementary file 1 [file biomolecules-13-01037-s001.zip › biomolecules-2391835-supplementary.pdf]

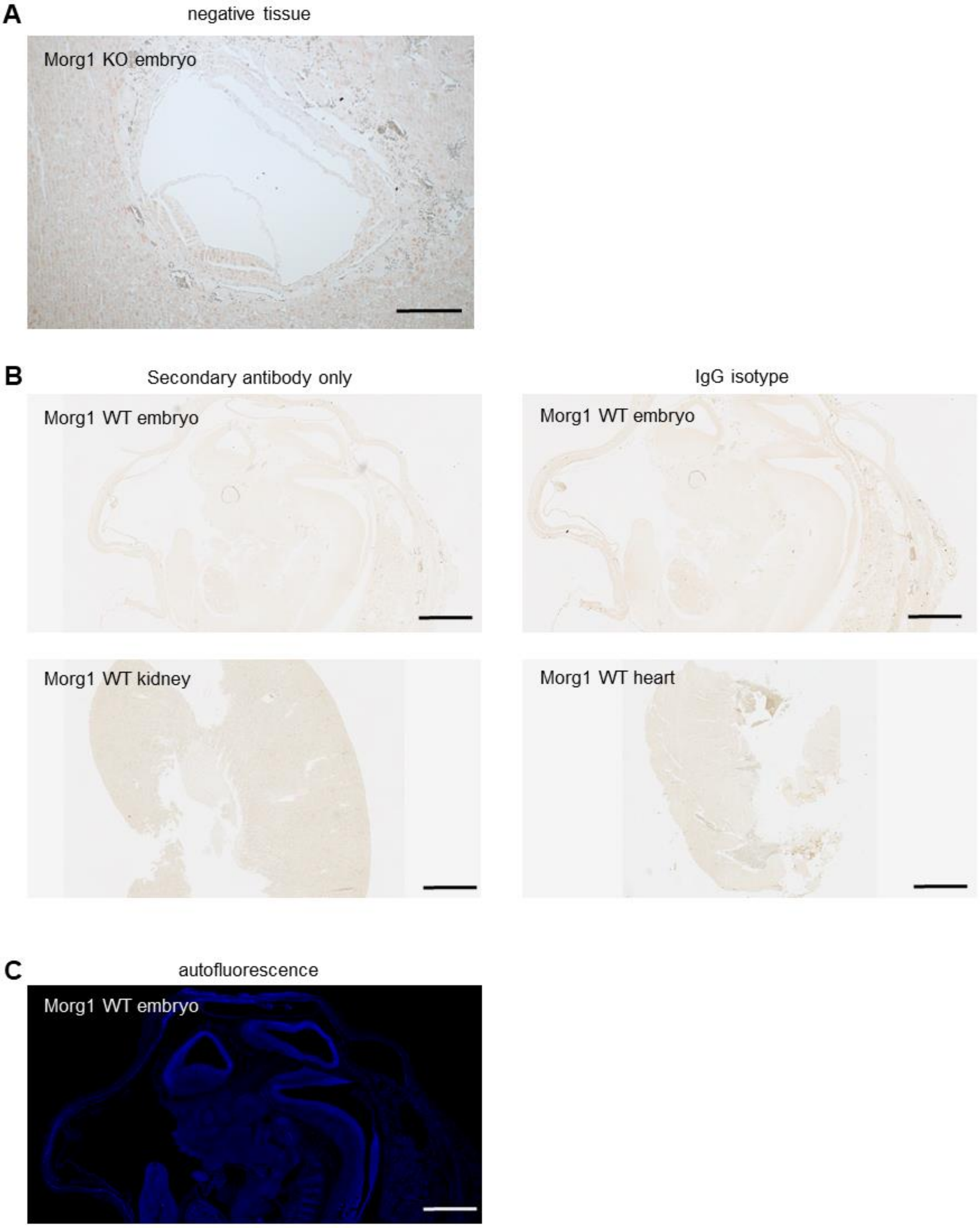

Figure S1. Control stains to assess the specificity of the antibody MORG1. (**A**) Representative image of "negative tissue" control on MORG1 knockout embryos. (**B**) Representative images of "secondary antibody only" and "isotype" control (IgG) on wildtype embryos and adult tissues. (**C**) Autofluorescence control of the mIFs on wildtype embryos. Scale bars = 500 μm.

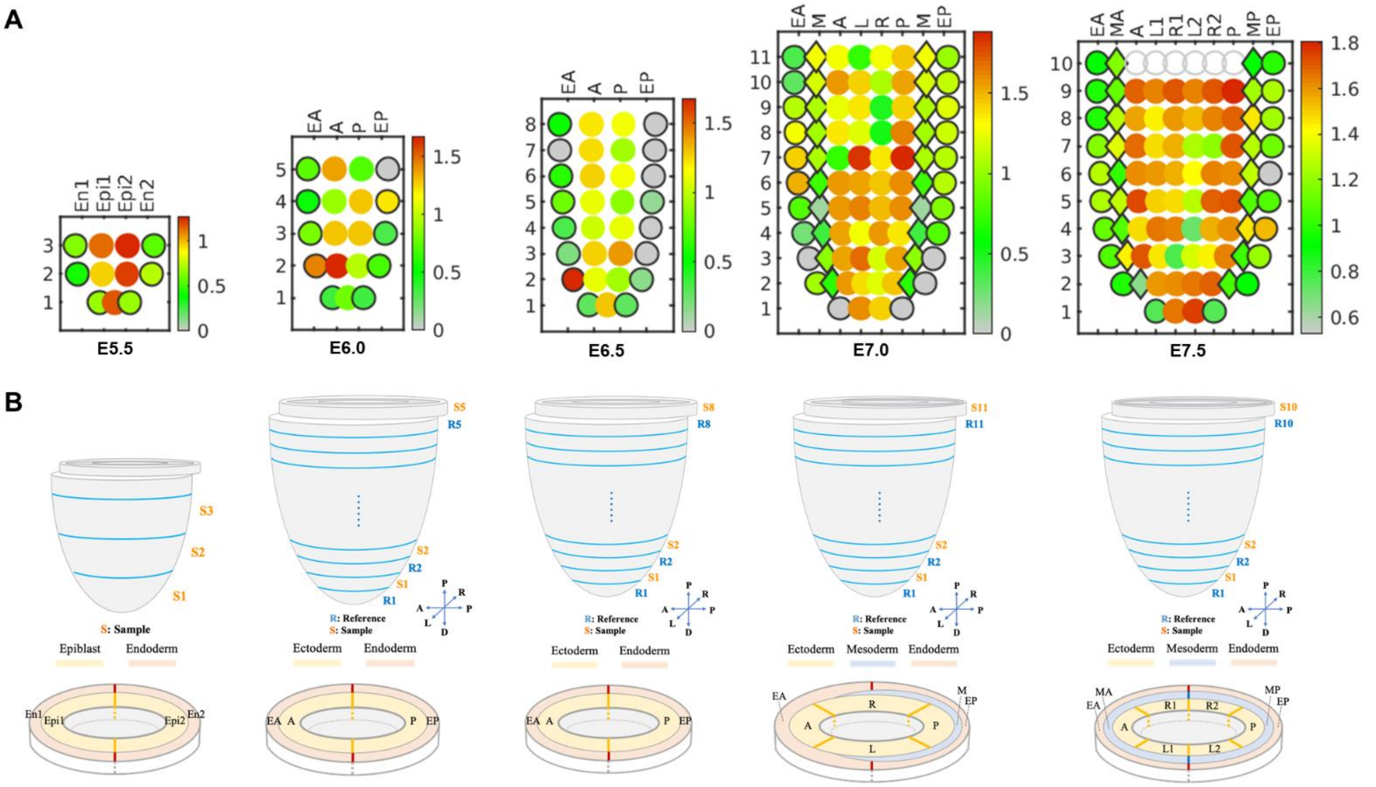

Figure S2. Spatiotemporal MORG1 transcription pattern of post-implantation mouse embryos generated by Geo-seq as annotated on the eGastrulation repository (http://egastrulation.sibcb.ac.cn/aHome/). (**A**) Corn plot representations of the spatial pattern of Wdr83 gene expression at indicated embryonic stages. Each dot in the plot represents the cell sample at the specific positional address, gene expression is color-coded as computed from the transcript counts in the RNA-seq dataset. Solid circles, epiblast–ectoderm; black outlined rhombus, mesoderm; black outlined circles, endoderm; grey hollow circles, no sample. (**B**) Schematic illustration of the Geo-seq procedure. A, anterior; P, posterior; L, left lateral; R, right lateral; L1, anterior left lateral; R1, anterior right lateral; L2, posterior left lateral; R2, posterior right lateral; Epi1 and Epi2, divided epiblast; M, whole mesoderm; MA, anterior mesoderm; MP, posterior mesoderm; En1 and En2, divided endoderm; EA, anterior endoderm; EP, posterior endoderm. For more details see references [45–47].
